# Addressing multiple long-term conditions in the undergraduate medical school curriculum: a focus group study

**DOI:** 10.1186/s12909-025-07484-1

**Published:** 2025-07-01

**Authors:** Steven T. R. Brown, Charlotte Rothwell, Deepika Manoharan, Bryan Burford, Gillian Vance

**Affiliations:** 1 School of Medical Education, Newcastle University, Newcastle-upon-Tyne, UK; 2 Population Health Sciences Institute, Newcastle University, Newcastle-upon-Tyne, UK

**Keywords:** Multimorbidity, Multiple long-term conditions, Medical education and training, Undergraduate, Postgraduate

## Abstract

**Background:**

Patients with a chronic physical disease accompanied by other disease types or biopsychosocial factors– multiple long-term conditions (MLTC)– represent a major and growing clinical challenge. 17% of the population of England are forecast to fit this definition by 2035. The aim of this study was to understand and explore desirable MLTC-related learning outcomes identified by newly graduated doctors in the UK.

**Methods:**

Focus groups were conducted across sites at two NHS trusts in Northern England with doctors in their second postgraduate year (Foundation Year 2 (FY2)). An iterative thematic analysis was applied to transcripts to identify and organise key themes.

**Results:**

Twenty-six participants across three focus groups reported their experience in primary and secondary care placements. The two overarching themes identified were: 1) ‘Practice needs’ for managing patients with MLTC. 2) ‘Education needs’ including limitations in undergraduate curricula. FY2s emphasised the concepts of uncertainty and complexity in practice, the variability of undergraduate learning experiences and gaps left by single-disease models of learning. Senior clinicians were highlighted as being key sources of support who modify learning experiences.

**Conclusions:**

Newly qualified doctors find MLTC care challenging and feel ill-prepared to manage patients on entering the medical workforce. Suggested improvements for undergraduate curricula include enhancing interprofessional methods of learning and ensuring consistency of exposure to, and focus on, MLTC patient-related complexity across undergraduate placements and curricula.

**Trial registration:**

Clinical Trial Number: Not applicable.

**Supplementary Information:**

The online version contains supplementary material available at 10.1186/s12909-025-07484-1.

## Background

The prevalence of multiple long-term conditions (MLTC, also known as multimorbidity, the co-occurrence of two or more chronic conditions in the same person) is a growing challenge for health and social care provision across the world [1–3]. Approximately 25% of adults in England are living with MLTC and estimates suggest that by 2035 this will rise to 68% amongst those aged 65 or older [4, 5]. However, multimorbidity is not limited to the elderly, and 30% of people living with four or more conditions are under the age of 65 [5]. A higher incidence is associated with lower socio-economic status [6]. Amongst individuals living in deprived areas, MLTC arises at an earlier age and has more complex presentations [7, 8]. 

The burden of disease associated with MLTC places a high workload on health and social care services [9, 10]. A recent review found that MLTC doubles the likelihood of a person using primary and secondary care services in the UK [11]. Patients often need more frequent and longer hospital stays and have an increased risk of emergency hospital admission or death [12, 13]. 

Even experienced GPs face challenges in MLTC care, relating to the prevalence of single-disease models of care, polypharmacy, challenges in managing prioritisation and shared-decision making [14, 15]. Doctors in training can struggle to manage the discordant yet potentially interacting symptoms [16], which can lead to missed opportunities for delivering high-quality patient-centred care [17]. 

The Department of Health and Social Care in England has placed empowering patients and the support of person-centred care at the heart of its strategic framework, with research into this patient cohort and their care a priority area across scientific and health research organisations [18, 19]. To improve services, education and training must also adapt to meet the needs of this growing population [20]. This was reinforced by the Chief Medical Officer for England in 2023, who noted that *“much of the medical profession is organised around single diseases or single organ systems in a way that is ill-suited to a future of increasing multimorbidity”.* [21 (p. 6)]

Despite this recognition, Lewis et al. [22] found sparse MLTC-related literature examining educational needs and interventions, and advocated for a recalibration of medical education to develop relevant management and communication skills. A focus on reforming undergraduate curricula may be the best route to providing all doctors with the foundational knowledge and skills required to manage these patients.

Whilst generalist skills are now increasingly incorporated into medical curricula, there remains significant opacity in how they are defined, valued and operationalised [23]. Most UK medical students are introduced to MLTC as an aspect of ageing and clinical frailty, as well as in general practice, but there is a lack of consensus on what needs to be learned, and how [24]. Curricula that incorporate Longitudinal Integrated Clerkships (LICs), where students spend longer periods of time in a particular clinical setting, may provide greater exposure to MLTC [25]. 

This study aimed to inform credible educational solutions for MLTC by considering the learning needs identified by doctors approaching the end of their first two years of postgraduate training (known as the Foundation Programme) in one region in England. Existing literature has focussed on the perceptions of more experienced clinicians, yet the perspectives of recent graduates on their knowledge, skills and practice has not been studied [16, 22, 26]. This may offer key insights into how curricula can be changed and optimised to better equip new doctors to manage patients with MLTC.

Accordingly, we explored the experience of doctors early in postgraduate training in relation to MLTC, any learning gaps they identified, and changes to undergraduate curricula they felt would have been beneficial.

## Methods

### Participants and recruitment

Participants were Foundation Year 2 doctors (FY2s) working in Trusts in the North East of England and North Cumbria. The Foundation Programme in the UK represents the first two years of post-graduation medical training, where doctors rotate through six four-month placements in medicine, surgery and community care. This is to provide a broad grounding of initial graduate medical experience and exposure. They practise under supervision of senior clinicians and take on increasing responsibility for patient care. This group was selected as they have had considerable exposure to patients with MLTC in both hospital and community settings but have graduated recently enough to reflect on their undergraduate training. The Foundation Programme curriculum also makes explicit the need to develop skills in managing acute illness in the context of multimorbidity [27]. 

Participants were invited by email to take part in a focus group, with a project information sheet sent by the local Foundation Programme administrator ahead of the session. No incentive was offered to attend, though lunch for participants was provided by the research team. Focus groups followed scheduled clinical teaching to maximise accessibility of participation.

### Data collection and analysis

Three focus groups were carried out in January 2022 in two NHS Trusts within the region. One focus group was hybrid (in-person and via Microsoft Teams) at one Trust (Trust A), while the other two took place in-person at another (Trust B). Sessions lasted for 60–90 minutes, and each was moderated by an early career researcher and experienced academic (GV, DM, SB, HA). Written consent was taken at the start of each focus group.

A semi-structured question guide was designed from the literature and in discussion within the research team (see appendix 1). Core questions included:

- *What have you found most challenging about looking after patients with MLTC?*
- *In what ways have you sought or been able to develop your clinical skills in MLTC?*

Additional questions and probes were responsive to group discussion and dynamics. Discussions were audio-recorded with participants’ consent and transcribed verbatim. Field notes were recorded as reflections by the focus group moderators.

A reflexive thematic analysis was carried out as described by Braun & Clarke [28]. Two researchers (CR, GV) listened to the recordings to check and familiarise themselves with the transcripts. All three transcripts were coded independently by CR and GV, who then discussed and agreed a shared coding framework. This was used to identify and organise themes through a series of iterations. Themes were discussed amongst the wider research group, and developed to provide a richer in-depth account to help answer the research questions in a reflexive manner. The researchers’ reflections were integrated into the outcome narrative.

None of the researchers had a teaching role with FY2 doctors at the time of the study. Three researchers were clinicians at different career stages and specialties, two at early career and one highly experienced researcher (GV, DM, SB), while two were researchers in medical education and social science research (CR, BB). A further highly experienced researcher facilitated one focus group and is acknowledged (HA).

## Results

Twenty-six FY2s, working in both hospital and community settings, took part in the study. Nineteen participants had graduated from Newcastle University Medical School, with the remainder graduating from other UK medical schools. Focus groups had 5, 12 and 9 participants, respectively, with overall gender mix 15 female and 11 male (see Table 1). Further demographic details were not collected.

**Table 1:** Focus group demographics

Focus Group (FG)	Hospital	Gender	Identifier
1 (Hybrid) (*n* = 5 participants)	A	4 females, 1 male	FG1
2 (*n* = 12 participants)	B	6 females, 6 males	FG2
3 (*n* = 9 participants)	B	5 females, 4 males	FG3

Analysis identified two overarching themes relating to the practice and learning context of MLTC. The first explored the everyday challenges identified by new doctors in caring for patients with MLTC. The second considered the nature of undergraduate and workplace-based education and training, associated shortfalls and potential changes.

### Practice needs: challenges of working with people with multiple long-term conditions

#### Complexity of patient management

Whilst exposure to patients with MLTC was commonplace, their clinical care was seen as complex and challenging. Participants described acute care management in the presence of MLTC comorbidities as being *‘completely different’* (FG3), giving examples of challenges in diagnosis, clinical decision-making, and planning, where there was a need to consider potential effects on other underlying conditions, as well as medication interactions.

*…my experience is more in general practice where patients come in feeling generally unwell. Which condition is causing it? Could be heart failure; you don’t know what medication to change*,* you don’t know what’s causing it. (FG3)*

Prompt and appropriate management might be further challenged by delays in important information-sharing between primary and secondary care.

The additional time needed to manage patients with MLTC was noted particularly by participants in primary care placements, who felt that the time allocated for a consultation was insufficient to assess a patient holistically. Whilst this reinforced prioritisation skills, it also left some trainees feeling dissatisfied with the care they provided. The brevity of placements, at four months duration, meant they often did not see the same patient again, limiting the experience of continuity of care.

*Without that time all you can deal with is the problem that they are coming with… and then put them to follow up if you identify something… ‘I need to see you again in a month’s time or in a week’s time because I want to talk about this because we haven’t had time to go through it’. (FG2)*

Complexity could make clinical work stressful, but also rewarding. The ability to treat acutely unwell patients successfully and enact patient-centred, holistic care were cited as positive experiences.

*I think it is very rewarding if you are able to sort them out acutely. Having considered when you step away from a complex patient*,* having managed to stabilise them. (FG1)*

#### Uncertainty, responsibility and risk

Complex clinical care caused feelings of uncertainty and worries about risk. Participants had been used to an algorithmic approach as medical students and found that they had to adapt to clinical situations that did not follow standard protocols. Polypharmacy was reported as a particular reason to be cautious in decision-making. Even when medicines were thought to be a potential cause of symptoms, they expressed worry about changing or stopping drugs for fear of destabilising the patient’s overall condition.

*And then it’s sort of*,* you know weighing up… Is it appropriate to keep that medication because it is helping her heart and the kidneys? But then it is…causing all these symptoms*,* causing her to fall over? (FG3)*

Participants were alert to the clinical consequences of making ‘wrong’ calls, whereby the patient could deteriorate because treatment either has not been aggressive enough, or adversely affects another system. Decisions can be high-stakes and further escalation may not be appropriate for some patients, placing heavy responsibility on newly graduated doctors.

*Actually*,* the worry is you don’t manage it well enough [so that they continue] deteriorating*,* or you make the wrong decisions and stuff you shouldn’t have done which sets their treatment back. (FG3)*

Adding to uncertainty around action, specialist involvement in patient care could deter FY2s from making management decisions without direction. FY2s did not feel empowered to alter plans, even if the clinical situation changed, because specialist input was seen as more valuable and qualified.

#### Recognising the limits of medicine

For many participants, the clinical experience of MLTC patients led to a shift in expectations about what medicine and inpatient care can– and should– achieve. These experiences challenged assumptions that all patients can be ‘cured’ and facilitated a shift in perspective from seeking to ‘fix’ to ‘optimise’.

*I think sometimes it is quite challenging… to fight the feeling that you just want to fix everything. And then I think you have just got to remember that you can’t fix everything…it is extremely difficult. (FG2)*

This may also challenge perceptions of what ‘being a doctor’ means, fostering an acknowledgement that MLTC patients may have different priorities, even if their conditions are the same. This supports the recognition of the need to provide individualised, patient-centred care.

*When it is somebody who has got multiple co-morbidities*,* it is not just about fixing everything*,* it is about achieving the best outcome for them. You know*,* you could have one patient with COPD*,* heart failure and CKD*,* and another one with exactly the same…but like I said their priorities are different. What they want out of life is different. (FG1)*

#### Complexity in communication

Having the ability to communicate with patients and their families is especially important for those with MLTC, as the information may be complicated, nuanced and uncertain. Whilst participants had some teaching on giving bad news as an undergraduate, they found it challenging to bring complexity into discussions with patients or relatives.

*We did quite a lot of teaching on SPIKES [A communication guide for difficult communication scenarios]* [29] *and how you break bad news […] complicated patients more frequently have bad news coming their way […] How much would be a good amount to go into here*,* how much is too little*,* are we going to document that part? (FG3)*

#### Discharging patients

The concept of the ‘safe discharge’ of MLTC patients was a specific context for communication and overarching management which participants found challenging. This requires them to make sure that the correct follow-up, support and medication is in place for patients, and that arrangements are timely, complete, and shared effectively.

*When you are discharging patients…you need more support. And it seems what support and how you access that support can vary on each ward and there doesn’t seem to be a very clear and coherent way…and what would be a safe discharge and what wouldn’t. (FG2)*

Participants highlighted the need for better information sharing between hospital teams, particularly in relation to medication management on discharge. They felt specialty teams and senior doctors needed to make plans clearer as they often felt left to make their own inferences. There was concern around medication follow-up and whose role this would be within the team.

*You are sat there as the junior doctor doing a discharge and then you are thinking*,* what do I restart […] who is actually overseeing this*,* I am kind of asking the GP to follow this up. (FG2)*

#### Working with different specialties

FY2s in the hospital setting have a key role in coordinating input from different specialties, services, and professionals. The challenges of norms of single-condition care were apparent in mixed messages from different specialties. Whilst specialists provided guidance on specific organ-based problems, it was often left to the FY2 doctor to reconcile different specialty views. A specialist might approach a patient’s symptoms and condition(s) differently to a generalist.

*What is frustrating is when you get different specialty [doctors] coming down and they are ‘right*,* change X*,* Y medications’*,* but they are very unclear plans…they expect you to implement that change and do all the leg work and think ‘Well can I actually do this in the context of the other medications?’ (FG2)*.

This meant that FY2s could be left confused as to how best to approach a patient. They would look for opportunities in ward rounds or multi-disciplinary team (MDT) meetings to seek a consensus view between different opinions.

*You then move to another department and the expectations there are totally different for exact same circumstances*,* the expectations from the seniors are totally different. And it is trying to then find balance of what each team would want you to do. (FG1)*

Patients who had a mental health problem alongside a physical condition brought another layer of challenge to interprofessional working. The interplay between physical and mental health was well recognised by participants, yet clinical teams often had different views on management. Participants reflected that medical and mental health services needed to take a more holistic view of MLTC patients to avoid circular discussions about a treatment plan.

*[When] you have someone with a long-term condition and a mental health problem. Because they are ill*,* then the mental health condition gets worse*,* but it means you can’t treat that until it [physical health problem] gets better. And you end up going to and fro between…services. (FG1)*

### Educational needs: what, when and how?

The second overarching theme related to perceptions of where and how learning linked to patients with MLTC takes place and its relevance for practice.

#### Curriculum design

Many participants reported that they had had little or highly variable exposure to patients with MLTC in their undergraduate training.

*I remember half of the long-term conditions was just having a 4-week placement somewhere and that experience varied massively. So*,* mine was not good at all […] it was kind of neuro-rehab and not the best clinical experience. Whereas I think others who were on care for the elderly placements had…beneficial and experiential learning. (FG2)*

Most participants had experience of a ‘spiral’ curriculum, with topics revisited and expanded from different perspectives across different years [30]. Participants indicated that this still took a single-system, single disease approach, and neglected the integration of information relevant for managing MLTC.

*It was very much you know ‘This is how you manage heart failure’. It was never ‘This is how you manage heart failure when the patient has got this*,* this and this’. (FG3)*

This lack of undergraduate exposure to MLTC impacted on postgraduate practice. Most participants felt underprepared to care for patients with MLTC on graduation and described current limitations despite their postgraduate experiences.

*I think if you start it [MLTC learning] in F1 it is too late. (FG1)*

#### Missed curricular opportunities

Participants commented on what they perceived as missed opportunities to learn about ‘MLTC medicine’ in undergraduate programmes. For example, they reported that teaching tended to focus more on the psychosocial impact of living with MLTC, rather than ‘medical’ aspects of management. Whilst participants recognised the importance of understanding the perspectives of patients and families, they felt they would have benefited from opportunities to explore the nuanced and complex management decisions required for this group of patients.

*When we were doing the prescribing tasks…even stuff like [analgesia if] someone is super co-morbid*,* their renal function is terrible– you don’t get given those scenarios as much. (FG1)*

However, there were examples of positive learning experiences through student assistantships and GP placements. Participants were able to recognise the importance of the MDT for patient care, and having opportunities to process how multiple factors influenced a patient’s wellbeing.

*My most useful encounter was during my final year GP placement*,* and it really changed my viewpoint…I would be having a parallel clinic with a GP and at times I’d take the history and come up with management plans by myself*,* trying to think through all the factors and then discuss it with the GP. (FG1)*

### Educational change: what would help students and trainees be more prepared?

Suggestions made by participants targeted both undergraduate and postgraduate settings and featured learning interventions relating to the processes and systems of care, which can help lead to effective MDT working in MLTC. These included collaborative learning with students from other health professions in order to address patient-related problems as well as utilising the expertise of others.

#### Simulation-based learning

Participants referred to the importance of including MLTC in simulation scenarios to allow students to practise and make any mistakes in a safe environment. Others suggested that more interprofessional education sessions, for example, with pharmacists, would help medical students understand their roles and responsibilities and how to work together.

*Sessions with pharmacists would be fantastic. I remember starting work and thinking wow pharmacists are amazing*,* they are incredibly helpful like I wish I knew more of what their role was sooner because they can just do so much to help you. (FG2)*

Further, communication skills training to support dialogue with senior doctors, other teams and professions would help graduates learn to navigate challenging interdisciplinary communication. Practicing communication on a wider variety of topics specific to MLTC needs, such as encouraging self-management, discharge planning and carer support, could be the focus of some sessions [22]. 

#### Practice-based learning

Many participants identified the importance of working with patients in the clinical environment. There was a common view amongst participants that learning is best gained through *‘on the job’* experience and no *‘algorithm’* exists to help with the complex scenarios they were encountering. Embedding these challenges within the context of real patients and workplace supervision was felt to be particularly useful.

*There is no amount of textbook training that can be given to fill that gap. (FG2)*

#### Support from healthcare professionals

Other doctors were most commonly referred to as supporting FY2 doctors’ learning experience. Participants referred to more senior trainees as a form of support, although other doctors, from FY2 peers to consultants, also shared their knowledge and experience of managing patients with MLTC.

*I think what was the most important at the point was just senior support probably. It just depended on who you worked with*,* whether you got that*,* but I think that was the most beneficial for our learning. (FG3)*

Participants identified other professionals who supported their learning, including nurses, pharmacists, occupational therapists, and physiotherapists. They commented that nurses often knew the patients well and gave crucial intelligence to support management decisions. Patients too were noted as an asset to learning as they provided not just a history of their treatment, but opinions on what mattered to them in their care.

## Discussion

This study has considered the views of doctors in the early stage of their postgraduate training around their experience of dealing with patients with MLTC and associated educational needs. Our data can inform changes to address limitations in undergraduate education, which may ultimately support improved care in this priority area [18]. Our participants, like all healthcare professionals, want to be able to provide holistic care for patients with MLTC. A patient-centred approach should be central to what is nurtured at practice level and developed at all stages of medical education.

Starting work as a new doctor is a particularly vulnerable transition, when sources of earlier support are often lost and new responsibility begins [31, 32]. Our findings suggest that among the new challenges faced is the management of patients with MLTC, with a perception that undergraduate curricula focus on neatly circumscribed systems and conditions and poorly represent the complexities of this patient group. They highlight a need for consistent and targeted exposure to MLTC patients before graduation. Specific challenges around polypharmacy and prescribing in episodes of acute deterioration were identified. Mistakes and near-misses are known to be key considerations for new graduates [33, 34]. The development of tailored teaching around prescribing in MLTC would be beneficial.

One key finding related to the caution and worry that FY2s feel about managing situations of clinical uncertainty. Monrouxe et al. [35] found similarly, and noted that senior clinicians do not trust the judgement of newly qualified colleagues and exclude them from decision-making, meaning that they can miss out on potential learning opportunities. Our data contrast with that study in that we identified that working as an intermediary between different specialist teams meant that management of MLTC-related complexity can often be *de facto* left to junior trainees. This places unwelcome responsibility on already anxious new doctors.

Literature suggests that any improvement in preparedness requires repeated exposure to similar situations [36], but the idiosyncratic presentation of MLTC means this may not always be possible. Further, while clinical algorithms can help guide management in acute scenarios, they do not offer the flexibility and responsiveness required for complexity in MLTC. Rather, multiple interacting patient, professional and contextual factors are weighed up by the doctor or interprofessional team, acknowledging the less predictable and non-linear effects of care interventions [37]. Whilst senior doctors provide important advice on management, there were often mixed messages between specialties, and support and learning were most commonly facilitated by more experienced doctors in training that often had a more generalist background. These findings call for better integration of MLTC management into undergraduate programmes to support learning about clinical complexity.

Another area of challenge related to aspects of communication, namely the need to adapt the nature of information and level of detail to individual patient and family needs. Participants felt that the best ways of conveying uncertainty had been a gap in their communication training, though felt comfortable dealing with some of the more emotionally challenging aspects of communication. Wider literature echoes these findings [33, 35]. 

### Supporting learning in practice

Optimising education for MLTC requires consideration of what, how and when skills relating to MLTC care should be learned. MLTC care is complex, involves multidisciplinary expertise and is highly individualised to the patient. Learning in MLTC can similarly be viewed through a complexity science lens. This conceptualises knowledge and skills as emerging from interprofessional collaboration as a result of dynamic relationships in a self-organising team [38]. Learning is collectively derived and owned. However, integrating students and new doctors into these teams can be challenging. Barriers include issues with continuity and time-constraints, whilst facilitators include appropriate expectation-setting and the support to contribute independently [39]. 

This balance between integration and agency in a social learning system is reflected in a Community of Practice, where learners engage in ‘legitimate peripheral participation’ as meaningful input into care in a supported manner [40]. Facilitating this during the peri-graduation period, spanning later undergraduate and early postgraduate training, may allow students to develop both formal and tacit knowledge about managing MLTC patients holistically in different settings, their role and contribution within the MDT, as well as skills in pragmatic shared decision-making and teamworking [40]. Introducing ways for undergraduates to participate in patient care, such as drafting prescriptions, may help them to develop profession-specific skills as well as consider how other team members can have beneficial input to their decisions [41]. 

Key to this may be the educational role of more senior doctors in training– knowledgeable ‘others’ who can enable learning experiences that help lift a doctor into greater levels of preparedness for the next time they face a similar situation [42]. Consideration of ways to recognise and develop these doctors so that they can best support situated learning of medical students in the workplace may be a fruitful area of further enquiry.

As well as supporting clinical ability, pastoral care of new graduates may require more attention in the immediate postgraduate environment. Although this was not explicitly asked about in focus groups, none of our participants mentioned seeking pastoral support despite sharing their anxiety about clinical risk and responsibility. However, if their clinical experiences misalign with their intent to deliver the best quality care, this poses a risk to their wellbeing as well as potentially fostering maladaptive and avoidant behaviours [43, 44]. 

### Curricula interventions

Curricular interventions which highlight the varying presentation of MLTC, acknowledge the challenges of management and incorporate MDT learning across specialties and professions need to be integrated throughout undergraduate education. The use of spiral curricula, where detail is added iteratively and focuses on case-based teaching, allows for the introduction of complexity and a deeper understanding of care for a particular patient group by revisiting cases with new knowledge, whilst incorporating early clinical experience to supplement this. MLTC and the needs of patients should be captured within these. Involving patients in curriculum development can help create more accountable and patient-centred curricula, as well as highlighting areas that may have been overlooked by education proviers [45]. 

Similarly, curricula for communication skills could graduate from simple interactions to communicating uncertainty across a programme, incorporating open acknowledgement of uncertainty, expectation setting and discussing possible outcomes and coping strategies [46]. The same strategies might also be useful in communicating uncertainty within the MDT.

More fundamentally, there is a conceptual, as well as educational question about how clinical reasoning for integration of complex information across systems or conditions is best learned, and whether it is a discrete skill, or situated in case specifics and explicitly linked to uncertainty [47]. The difficulty our participants faced suggests that complexity changes the nature of the skill. Clinical reasoning in MLTC is a not a simple, deductive process. It prompts the inclusion of non-deductive factors such as uncertainty tolerance, prior experience and knowledge of the patient into a complex cognitive process that can have unexpected outcomes [37]. Literature examining uncertainty tolerance has found dynamic relationships between cognitive, emotional and behavioural responses to uncertain clinical situations, therefore curricula must encourage students to explore these psychological phenomena, learn to navigate complexity in clinical reasoning and support the development of useful adaptive behaviours [44]. The emphasis of case-based learning and simulation could be adjusted, and this could also be factored into placements aiming to build self-reflective and pragmatic patient care skills and cognitive processes.

Our participants often did not see the same patient more than once and felt this hindered their learning from patient experiences with MLTC. One curricular solution to this could be found in a Longitudinal Integrated Clerkship, whereby medical students have an extended period in a workplace [48]. This can provide greater opportunities for continuity and experience managing a patient workload under supervision [25]. LICs constructed to explicitly support learning around MLTCs could provide benefit if designed appropriately with a supportive setting and supervisory team [49]. 

Any solution to the question of doctors’ under-preparedness to manage MLTC requires careful planning as to the conceptual and knowledge-based scaffolding required to develop safe heuristics of practice. Targeted teaching to highlight the distinctive challenges of MLTC may provide a grounding for learning to prepare new doctors for seeing them in practice but cannot provide the practical experience of all idiosyncrasies. There is often hesitancy in undergraduate curricula to present the complexity and messiness of clinical practice [50]. More radical approaches which expose medical students to that complexity, safely, and earlier, may help ensure they are prepared for the growing numbers of complex patient presentations in real-world practice. These may include supervising student interaction with MLTC patients, allowing them to independently explore issues and supporting reflection and debriefing [51]. Interprofessional activities might also be constructed with creative assessment methods, supporting the emergence of collective multidisciplinary knowledge [52]. 

### Limitations

The study was conducted in one area of England within two trusts only, and most participants had attended the same medical school, meaning differences in undergraduate curricula and experience between institutions will not have been fully represented. However, as all undergraduate curricula in the UK must meet the same GMC requirements [53], a degree of equivalence is likely for graduates of other UK institutions.

A further limitation was the use of focus groups only. Participants can feel reluctant to share thoughts or experiences when surrounded by peers who they feel may judge them negatively for expressing uncertainty in their own practice. Therefore, the invitation to participants emphasised the purpose of improving medical education by identifying participant needs and issues. Two facilitators, of which at least one was an experienced researcher, conducted each focus group and were directed to seek input from all participants in an exploratory manner to facilitate constructive discussion [54]. A hybrid format was employed to maximise attendance given Hospital Trust A was in a more rural and less accessible location. Moderators were encouraged to invite participation explicitly from those attending via Microsoft Teams and the session was arranged around a screen. This was to mitigate the bias likely to arise from the lack of physical presence as well as the possibility of reduced richness of data from these participants [55]. 

## Conclusions

Patients with MLTC pose management challenges which generate uncertainty and unease for new doctors. With little current evidence on how best to educate our future doctors in this area, this study provides valuable insights into the core issues that medical education must address. Future interventions should be grounded in the realities of the workplace, emphasising the central role of the MDT, continuity and supported engagement and practice with both real and simulated patients. They should also recognise and support the educational function of senior clinicians to optimise ongoing workplace-based learning around MLTC.

## Electronic supplementary material

Below is the link to the electronic supplementary material.

Supplementary Material 1

## Data Availability

The datasets generated and/or analysed during the current study are not publicly available due to their collection as audio recordings and verbatim transcripts which could be identifiable, but are available from the corresponding author on reasonable request.

## Abbreviations

FG: Focus Group
FY2: Foundation Year 2 Doctor
GMC: General Medical Council
GP: General Practitioner
LIC: Longitudinal Integrated Clerkship
MDT: Multidisciplinary Team
MLTC: Multiple Long–Term Conditions
NHS: National Health Service
SPIKES: Setting, Perception, Invitation, Knowledge, Emotion, Summary
UK: United Kingdom
