# Supplementary material for: Addressing multiple long-term conditions in the undergraduate medical school curriculum: a focus group study
Source: BMC Med Educ. 2025 Jul 1;25:987. doi: 10.1186/s12909-025-07484-1 (PMC12220220; doi:10.1186/s12909-025-07484-1)
Supplement: Supplementary file 1 — Supplementary Material 1 [file 12909_2025_7484_MOESM1_ESM.docx]

# Appendix 1

## Focus Group Interview Guide/Question Schedule

1. [clarify definition and understanding of the subject area] What do you understand by the term ‘multi-morbidity’? (one or two words or phrase on post-it)
2. [nature / complexity of patient encounters] What experience have you had of seeing patients with MLTC/multimorbidity?
   - What types or combinations of condition have you seen? In what setting?
   - Were these new or existing patients?
   - Did you have repeated contact with these patients?
3. [clinical / educational needs in practice] a) What have you found most challenging about seeing/managing patients with MLTC/multimorbidity? b) What have you found most rewarding?
   - How did you approach these patients differently, if at all?
   - Did their management/referral differ in clear ways from other patients?
4. [involving others; working in a team; other educational needs in practice] What do you think the doctor’s role is in seeing/ managing patients with MLTC/multimorbidity?
   - Who else (eg, other HCP) have you found to be important in the management of these patients?
   - In what ways have you worked with these professionals in the care of patients with MLTC?
   - Has this highlighted any additional skills you need?
5. [educational supports to deal with MLTC] In what ways have you sought / been able to develop your management skills in MLTC/multimorbidity? (What have been your educational ‘go to’ sources?)
6. [undergraduate preparation and gap] What, if any, aspects of undergraduate medical education had prepared you for that experience?

- Were there any learning outcomes/experiences in your undergraduate programme that have helped you identify or manage patients with MLTC?
- Was MLTC explicitly defined in your UG programme?

1. [undergraduate preparation and gap] What changes would you make to your undergraduate curriculum to better prepare you?

- Do you think undergraduate medical education is an appropriate time to address this topic?

1. Are there any other points you would like to make in relation to this area that you feel we haven’t covered?
